# A Single-Center Review of Facial Fractures as the Result of High-Speed Projectile Injuries

**Published:** 2018-04-09

**Authors:** Farrah C. Liu, Jordan N. Halsey, Ian C. Hoppe, Frank S. Ciminello, Edward S. Lee, Mark S. Granick

**Affiliations:** ^a^New Jersey Medical School, Rutgers Biomedical Health Sciences, Newark; ^b^Division of Plastic Surgery, Department of Surgery, New Jersey Medical School, Rutgers Biomedical Health Sciences, Newark; ^c^Department of Plastic Surgery, Craniofacial and Pediatric Plastic Surgery, Hackensack University Medical Center, Maywood, NJ

**Keywords:** facial fractures, facial injury, gunshot injuries to the face, high-velocity facial trauma, projectile injuries

## Abstract

**Purpose:** Gunshot injuries to the face that result in fractures of the underlying skeleton present a challenge in management. The goal of this study was to evaluate patterns of facial fractures as a result of gunshot injuries and strategies for management. **Methods:** A retrospective review of facial fractures resulting from gunshot injuries in a level 1 trauma center was performed for the years 2000 to 2012. Data were collected for patient demographics, fracture distribution, concomitant injuries, and surgical management strategies. **Results:** A total of 190 patients sustained facial fractures from a gunshot injury. The average age was 29.9 years, and 90% were male. Sixteen injuries were self-inflicted. The most common fractures were of the mandible and the orbit. Uncontrolled hemorrhage was noted on presentation in 68 patients; 100 patients were intubated on arrival. The average Glasgow Coma Scale score on arrival was 11.9. Concomitant injuries included skull fracture, intracranial hemorrhage, and intrathoracic injury. Surgical management was required in 89 patients. Nine patients required soft-tissue coverage. Thirty patients expired. **Conclusion:** Gunshot injuries to the face resulting in fractures of the underlying skeleton have high instances of morbidity and mortality. Life-threatening concomitant injuries can complicate management of facial fractures in this population.

Gunshot injuries to the face often present as complex reconstructive challenges with regard to acute surgical treatment and long-term management. Prompt surgical restoration of soft tissue and bony stabilization of facial fractures are vital to achieve an optimal functional and aesthetic outcome.^1^^,^^2^ Early intervention can often prevent bone misalignment, resorption, and scarring, as well as preserve well-vascularized soft tissue that is integral to avoidance of further bone loss and infection.^3^^,^^4^ Comprehensive imaging is imperative, especially when other injuries are present, to avoid inaccurate diagnosis of underlying facial fractures and delay of timely and appropriate management.^5^^,^^6^ Examples of the extent of injury seen in these patients are demonstrated in Figures 1*a*–1*c*.

While primary surgical bony and soft-tissue reconstruction is recommended, concomitant injuries that take precedence can hinder such timely approaches.^7^ Penetrating facial gunshot injuries tend to involve multiple organ systems and present with other complications such as airway compromise and uncontrolled hemorrhage that may delay management.^8^^-^10 Aside from delayed timing, inappropriate selection or application of surgical technique may lead to further complications that increase postoperative morbidity, length of hospital stays, and cost of treatment.^11^ Even in cases of adequate repair of facial injuries, foreign bodies retained in the tissue related to the initial gunshot injury can lead to infection and delayed wound healing.^12^

The objective of this study was to examine facial fractures as a result of gunshot injuries in order to elucidate demographics, patterns of injury, concomitant injuries, fracture management, and soft-tissue management at a level 1 trauma center.

## METHODS

Following institutional review board approval, all radiologically diagnosed facial fractures as a result of gunshot injuries at a level 1 trauma center (University Hospital, Newark, NJ) between January 2000 and December 2012 were reviewed on the basis of *International Classification of Disease, Revision 9* (*ICD-9*) codes. The results were further categorized by location of fractures, concomitant injuries, services consulted, and patient demographics during initial assessment of the trauma. Information on surgical management strategies and total length of hospital stay was also collected. The Glasgow Coma Scale (GCS) was used to assess the level of consciousness on presentation. For modality of injury, low-velocity weapons were classified by projectiles traveling slower than 1200 ft/s, whereas high-velocity weapons produced projectiles traveling faster than 2000 ft/s.^13^ Patients who were admitted with uncontrolled facial hemorrhage, defined as the need for some form of intervention to arrest bleeding (including packing, interventional radiology embolization, and direct vessel ligation), were specifically noted and treated accordingly.

## RESULTS

A total of 3147 facial fractures were treated at University Hospital from 2000 to 2012, 190 of which were identified as the result of gunshot injuries. A description of findings is included later. Three-dimensional computed tomographic reconstruction imaging of various patients can be visualized in Figures 1*a*–1*c*.

### Demographics/mechanisms of injury

The average age of the patients was 29.9 (range, 14-81) years, with a strong male predominance (91.6%). Sixteen (8.4%) of the injuries were self-inflicted. Ninety-seven percent of the weapons used were determined to be low velocity, whereas the remaining 3% were intermediate velocity, and none were high velocity.

### Distribution of fractures

Of the 386 fractures recorded in these patients, the most common fractures were of the mandible (23.3%). Fractures of the orbit (20.5%) were the next most common, followed by the zygoma (13.7%) and the nasal bone (12.2%). Distribution of fractures is demonstrated in Figure 2.

### Concomitant injuries

On admission, the average GCS score was 11.9 (range, 3-15). During, or prior to, arrival in the trauma bay, 100 (52.6%) of the patients were intubated and 68 (35.8%) presented with uncontrolled facial hemorrhage. The most common concomitant injury was a skull fracture (31.6%), closely followed by intracranial hemorrhage (29.5%), intrathoracic injury (18.9%), cervical spine fracture (14.2%), pelvic/thoracic fracture (11.6%), long bone fracture (11.1%), intra-abdominal injury (5.3%), and lumbar spine fracture (4.2%). Of the majority of patients (n = 176) admitted to the hospital, 118 were admitted to an intensive care setting. Distribution of concomitant injuries is demonstrated in Figure 3.

### Services consulted

Fracture cases were managed by plastic surgery, otolaryngology, oral and maxillofacial surgery (OMFS), and ophthalmology. Distribution of services consulted is demonstrated in Figure 4.

### Fracture management

Surgical management of fractures in the operating room was required in 89 of the 190 patients, the majority of which underwent debridement of devitalized tissue with or without rigid internal fixation. Of the patients treated, 139 (77.7%) were able to be managed conservatively with maxillomandibular fixation whereas 32 (17.9%) required internal fixation and 8 (4.5%) required external fixation (Fig 1*c*). All other patients underwent some form of bedside irrigation with conservative debridement.

### Uncontrolled facial hemorrhage

Of the patients with uncontrolled hemorrhage at presentation, 71% were treated with conservative measures including packing and direct pressure, whereas 22% underwent interventional radiology embolization and 7% required operative vessel ligation.

### Soft-tissue management

Only 9 patients required local or regional flaps for soft-tissue coverage. In 1 patient, enucleation and orbitotomy were required to remove the bullet casing and a tarsorrhaphy and a sliding lower lid flap were utilized to close the wound.

### Hospital stay/mortality

The mean hospital length of stay was 12.9 (range, 0-228) days. Thirty patients expired. Patients who expired were significantly older than patients who survived, aged 36.4 and 28.3 years, respectively (*P* < .05). Patients who expired also had a significantly lower GCS score on arrival than patients who survived (5.9 and 13.1, respectively) (*P* < 0.05).

## DISCUSSION

### Demographics/mechanism of injury

There was a heavy male predominance (91.6%), with low-velocity weapons most commonly used. Low-velocity weapons are likely easier to access and to be utilized in a heavily populated urban environment such as Newark, NJ. High-velocity weapons such as rifles can result in a much higher degree of damage, as the fragmented bone from the initial impact can further destroy the adjacent soft tissue.^12^ Thus, while low-velocity gunshot fractures are difficult to treat, they are more manageable than intermediate- or high-velocity fractures.

### Distribution of fractures

The mandible was the most commonly fractured bone in this study, followed closely by the orbit, zygoma, and nasal bone. Other studies support this finding.^14^^,^^15^ It is unclear whether this is due to the situations under which these injuries occur or to the location and composition of these specific facial structures.

### Concomitant injuries

The most common concomitant injuries were skull fractures, closely followed by intracranial hemorrhage. The lower trunk, including the intra-abdominal region and thoracic and lumbar spine, suffered the least injury. These injuries often take precedence to facial fractures, especially those admitted with a depressed GCS score. Seven of the 17 patients with a GCS score of 3 expired.

In these cases, management of facial fractures is delayed, which can hinder the final aesthetic and functional outcomes by affecting both soft-tissue and bone healing. Infection is also more likely to develop because of these wounds remaining insufficiently debrided secondary to the management of more life-threatening injuries.

### Services consulted

Facial fractures from gunshot wounds often present as complex injuries that require interspecialty collaboration. At our institution, these cases are managed by plastic surgery, otolaryngology, OMFS, and ophthalmology. It was surprising how infrequently plastic surgery was consulted, especially in light of the often-associated soft-tissue injuries. This may represent an institutional bias to place more emphasis on the bony injury and is something that we are investigating to attempt to improve the management of these patients at our institution.

### Hospital stay/mortality

The mean hospital length of stay was 12.9 days and ranged from 0 to 228 days. Patients with a depressed GCS score were more likely to expire, which is intuitive and is likely a surrogate for the severity of systemic injury sustained.

### Soft-tissue management

Only 9 patients of the 190 treated required local or regional flaps for soft-tissue coverage. This can be attributed to the predominance of low-velocity ballistic weapons, as intermediate- and high-velocity weapons often cause more soft-tissue destruction.

### Fracture management

The majority of patients were treated conservatively with maxillomandibular fixation, whereas the remaining required internal or external fixation. The fact that the majority of patients required only conservative treatment may be partially explained by the nature of the injury. Most were attributed to low-velocity handgun injuries, which cause considerably less soft-tissue loss and comminution of bone.

In all cases, irrigation and debridement were performed to remove devitalized tissue and foreign bodies from the wound. Maxillomandibular fixation was frequently used because of the presence of concomitant injuries, comminution of bone, and perceived difficulty obtaining a satisfactory result with rigid fixation. In cases where there was a significant loss of bone, external fixation was utilized. Tracheostomy was performed when there was a concern for airway protection or based on discussion with colleagues in trauma surgery.

## CONCLUSION

Patients with facial fractures resulting from gunshot injuries often have complicated presentation and have unique concerns for fracture management. These cases are further complicated by concomitant injuries that may require more immediate attention. A multidisciplinary approach is integral for optimal results. Once acute issues are resolved, appropriate soft-tissue management and skeletal stabilization of fractures are essential for proper management of gunshot injuries to the face to minimize secondary deformities. Removal of foreign objects and fracture fragments, as well as adequate debridement of nonviable soft tissue, should always be performed prior to any reconstructive procedures to prevent secondary complications.

## Figures and Tables

**Figure 1a:**
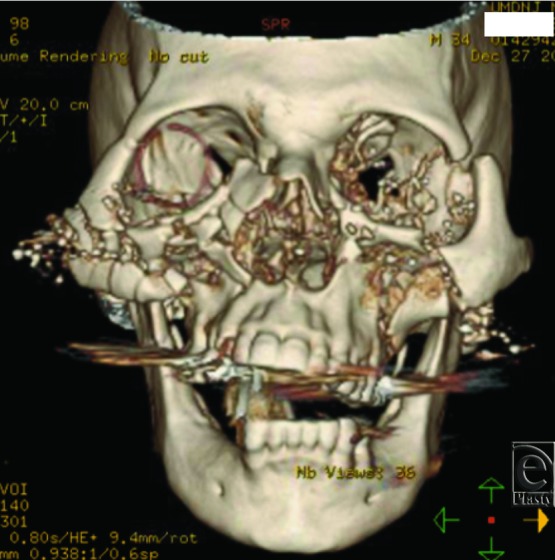
Three-dimensional reconstructions of projectile injuries to the craniofacial skeleton.

**Figure 1b:**
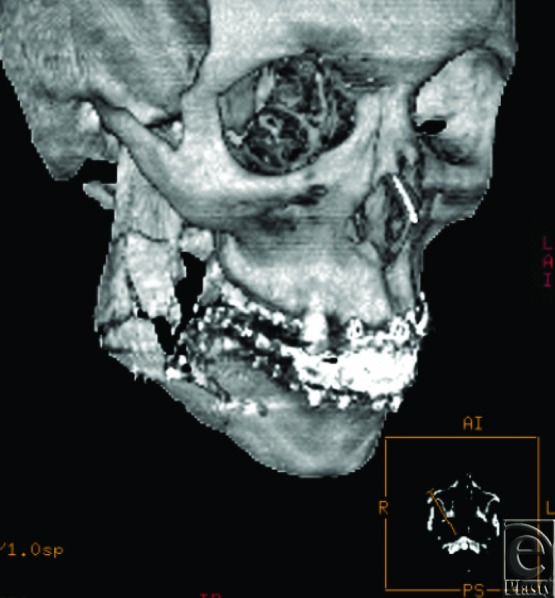


**Figure 1c:**
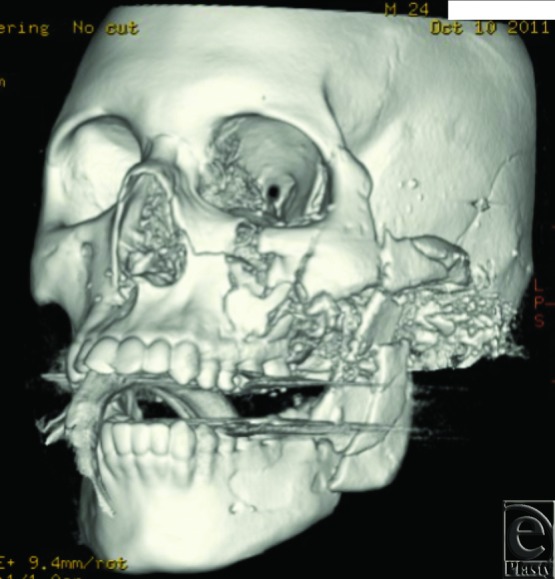


**Figure 2:**
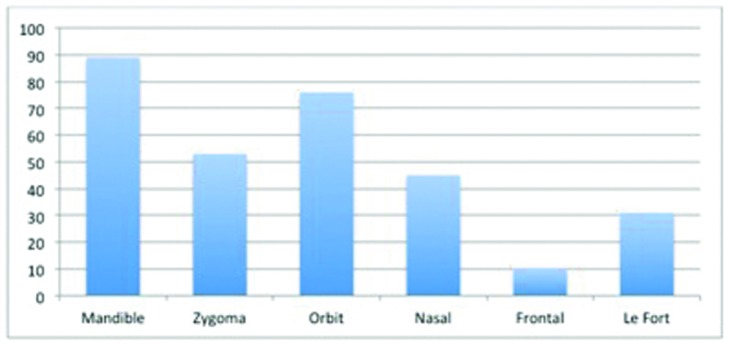
Distribution of fractures by anatomical site.

**Figure 3:**
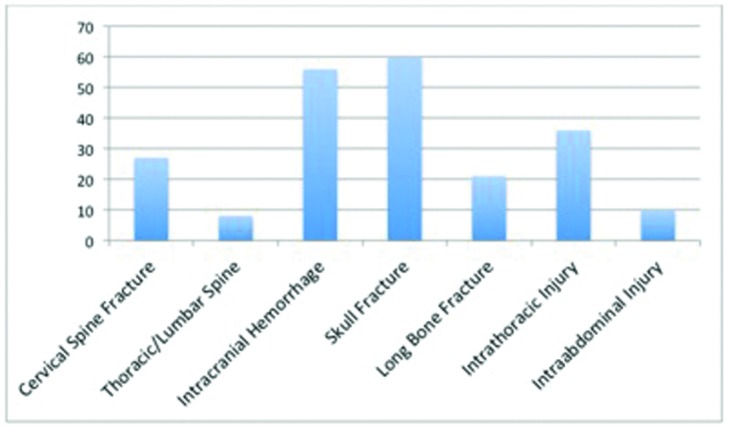
Distribution of concomitant injuries.

**Figure 4:**
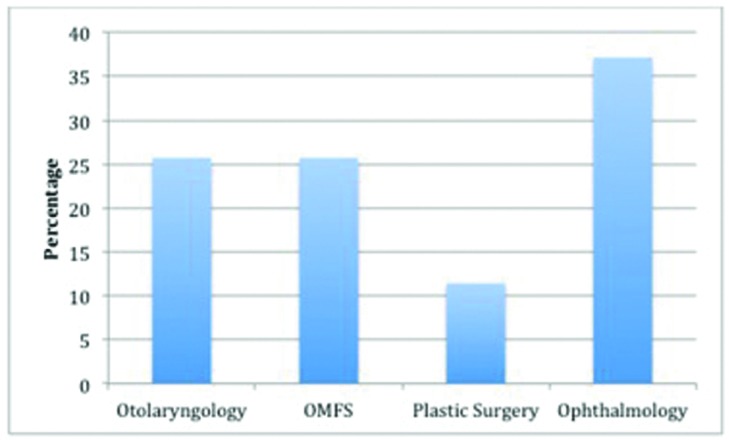
Distribution of services consulted.
